# Pain Characteristics and Progression to Sarcopenia in Chinese Middle-Aged and Older Adults: A 4-Year Longitudinal Study

**DOI:** 10.1093/gerona/glae080

**Published:** 2024-03-12

**Authors:** Jintao Chen, Liying Yan, Jingjing Chu, Xinyi Wang, Zherong Xu

**Affiliations:** Department of Geriatrics, The First Affiliated Hospital, Zhejiang University School of Medicine, Hangzhou, China; Department of General Practice, The Second Affiliated Hospital, Jiangxi Medical College, Nanchang University, Nanchang, China; Department of Medical Records, The First Affiliated Hospital, Zhejiang University School of Medicine, Hangzhou, China; Department of Geriatrics, The First Affiliated Hospital, Zhejiang University School of Medicine, Hangzhou, China; Department of Geriatrics, The First Affiliated Hospital, Zhejiang University School of Medicine, Hangzhou, China; Key Laboratory of Frailty Research in Older People, Zhejiang Provincial Administration of Traditional Chinese Medicine, Hangzhou, China; (Medical Sciences Section)

**Keywords:** CHARLS, Pain characteristics, Sarcopenia

## Abstract

**Background:**

It is imperative for public health to identify the factors that contribute to the progression of sarcopenia among middle-aged and older adults. Our study aimed to investigate the association between pain characteristics and the progression to sarcopenia and its subcomponents among middle-aged and older adults in China.

**Methods:**

We included 5 568 participants from the China Health and Retirement Longitudinal Study. All participants completed assessments for pain characteristics and sarcopenia. Pain assessment included pain status (baseline pain, incident pain, and pain persistence) and pain distribution (single-site pain and multisite pain) using a self-report questionnaire. Diagnosis of sarcopenia followed The Asian Working Group for Sarcopenia 2019 consensus. The odds ratios (ORs) and 95% confidence intervals (CIs) were obtained by logical regression analysis.

**Results:**

Participants who reported baseline pain, multisite pain, pain persistence, or multisite pain persistence were more likely to progress to sarcopenia than those without pain, with ORs of 1.33 (95% CI: 1.08–1.65), 1.44 (95% CI: 1.15–1.80), 1.63 (95% CI: 1.23–2.14), and 1.59 (95% CI: 1.19–2.11), respectively. Even after adjusting for other covariates such as gender, age, residential area, education level, marital status, smoking, alcohol consumption, comorbidities, and falls, these associations remained significant. Additionally, pain persistence and multisite pain persistence were significantly associated with low grip strength and clinically meaningful Short Physical Performance Battery decline, but not with low muscle mass.

**Conclusions:**

Our study showed that pain, especially pain persistence, was closely correlated to the increased risk of progression to sarcopenia in Chinese middle-aged and older adults.

Increasing evidence suggests that when individuals reach their mid-fifties, muscle mass tends to decrease by approximately 1% to 2% annually, accompanied by a decline in muscle strength, which diminishes at a pace ranging from 1.5% to 5.0% per year (1). This age-related muscle mass decrease, known as “sarcopenia,” carries significant physiological and clinical implications (2,3). Many studies have demonstrated close correlations between sarcopenia and negative consequences such as falls, disability, and premature mortality (3–5). Currently, sarcopenia affects over 50 million individuals worldwide, with projections suggesting an impact on more than 200 million people in the next 40 years (6). Therefore, it is imperative for public health to identify factors contributing to the progression of sarcopenia in middle-aged and older people.

Numerous studies have identified several factors, such as aging, poor nutrition, lack of physical activity, dental conditions, and diabetes, independently associated with sarcopenia (7). However, some potential risk factors, like pain, remain underexplored. Pain, an unpleasant sensory and emotional experience often indicative of tissue damage (8), is prevalent among middle-aged and older adults. According to statistics from the China Health and Retirement Longitudinal Study (CHARLS) data, about 28.62% to 37.27% of middle-aged and older people in China suffer from pain in their daily lives (9). Evidence suggests pain can precipitate negative outcomes, including sedentary behavior, malnutrition, loss of daily functioning, and frailty (10–12), which may also contribute to the onset and progression of sarcopenia (13). However, the association between pain and sarcopenia remains inconclusive in existing literature. For instance, one cross-sectional study showed that low back pain was related to frailty, but not to sarcopenia (14). Conversely, the results of 2 recent longitudinal studies and a meta-analysis reported that pain may increase the risk of sarcopenia (15–17). Nonetheless, there are some limitations in these studies. For instance, the study by Lin et al. had only 1 year of follow-up and a limited sample size, so the results were not representative enough (16). The study by Veronese et al. used the anthropometric formula that was validated only in the United States (15). Considering the significant differences in muscle mass between different races (18), these limitations affect the general applicability of this study about the role of pain in sarcopenia (15). Therefore, prospective cohort studies with large samples are very necessary to fully investigate the association between pain and sarcopenia.

In this study, we conducted a longitudinal analysis using nationally representative data to investigate the potential relationship between pain characteristics and sarcopenia and its subcomponents in Chinese middle-aged and older adults to clarify whether pain is a risk factor for progression to sarcopenia status. This will help clinicians improve measures for the prevention and intervention of sarcopenia, ultimately helping to preserve physical function and quality of life in aging populations.

## Method

### Setting and Participants

The China Health and Retirement Longitudinal Study is a prospective cohort study that encompasses a population-based sample, targeting individuals aged 45 years and older in China. It employs one-to-one interviews and structured questionnaires to gather high-quality data for assessing the aging population’s status and promoting interdisciplinary aging research. The study was performed in compliance with the Helsinki Declaration and received approval from the Biomedical Ethics Committee of Peking University (IRB00001052-11015). Informed consent was obtained from all participants via a signed consent form prior to the commencement of the study. The initial survey was conducted in 2011 with over 17 000 respondents participating. Subsequent follow-ups were scheduled every 2–3 years. Comprehensive details on the CHARLS protocols are available in prior publications (19).

Our study utilized data from the CHARLS 2011 baseline survey and 2015 follow-up survey. Of the initial cohort, 14 574 participants were present at both the baseline and the follow-up 4 years later. Exclusion criteria included individuals younger than 50 years at baseline survey (*n* = 3 280), those with incomplete data on pain characteristics (*n* = 110) or sarcopenia status at baseline survey (*n* = 2 795), and participants with sarcopenia at baseline survey (*n* = 1 361). Additional exclusions at follow-up pertained to missing data on pain characteristics (*n* = 95) or sarcopenia status (*n* = 1 365). Ultimately, the analysis encompassed 5 568 subjects (Supplementary Figure 1).

### Measurement of Sarcopenia Status

Sarcopenia was evaluated following the flow chart used in the 2019 revised recommendations of the Asian Working Group for Sarcopenia (7). Evaluations included physical performance, muscle strength, and appendicular skeletal muscle mass (ASM).

The handgrip strength of each participant’s dominant and nondominant hands was measured using a YuejianTMWL-1000 dynamometer (Nantong Yuejian Physical Measurement Instrument Co., Ltd., Nantong, China). Each participant’s dominant and nondominant hand grip strengths were measured twice. We selected the 2 larger values from the 4 measurements and then calculated the average of the 2 larger values as the grip strength value for this participant. Low muscle strength was defined by cutoff values of less than 18 kg for females and 26 kg for males (7).

The Short Physical Performance Battery (SPPB) was utilized to evaluate physical performance, and it consisted of 3 components: standing balance, walking speed, and a 5-repetition chair stand test (7). The standing balance test required participants to hold their feet in a semi-tandem, side-by-side, and tandem position for 10 seconds each. Walking speed required participants to walk 2.5 m twice at a normal speed and record the time they completed each time. The average time of 2 times was taken to calculate the normal walking speed. The 5-repetition chair stand test required participants to stand up 5 times in a row while crossing their arms in front of the chest, and the completion time was recorded. An SPPB score ≤9, 5-repetition chair stand test time ≥12 seconds, or walking speed <1.0 m/s were indicative of low physical performance (7).

The appendicular skeletal muscle (ASM) was estimated by a previously validated physical measurement formula in Chinese residents (20): ASM = 0.107 × height (cm) + 0.193 × weight (kg) − 0.037 × age − 4.157 × gender − 2.631, with gender coded as 0 for females and 1 for males. This formula has shown high concordance with dual x-ray absorptiometry measures (20,21). The height-adjusted muscle mass (ASM/Ht^2^) was computed by dividing ASM by the square of the individual’s height in meters to adjust for the effect of height on muscle mass. Similar to earlier research, low muscle mass was identified as the lowest quintile of the ASM/Ht^2^ according to sex-stratified values among the study population (22–24). Therefore, to identify individuals with low muscle mass in this study, ASM/Ht^2^ values of <5.19 kg/m^2^ in females and <6.98 kg/m^2^ in males were used as cutoff points.

### Assessment of Pain Characteristics

Pain characteristics were assessed based on the following self-reported questions: “Are you often troubled with any body pains (‘no’ or ‘yes’)? On what part of your body do you feel pain? Please list all parts of your body where you are currently feeling pain (head, neck, chest, stomach, shoulder, back, waist, buttocks, arm, leg, knees, wrist, fingers, ankle, toes).” According to the pain characteristics assessment report at the baseline survey, we divided the pain status into no pain and baseline pain. We divided the pain distribution into no pain, single-site pain, and more than one site of pain (multisite pain). Similar to other observational pain studies (25,26), by combining the pain characteristics assessment reports at the baseline survey and the follow-up survey 4 years later, we further categorized pain status into the following categories: (1) reporting no pain at both baseline survey and follow-up survey (no pain); (2) reporting pain at the same site at both baseline survey and follow-up survey (pain persistence); and (3) reporting pain at baseline survey but no pain at follow-up survey, or reporting no pain at baseline survey but pain at follow-up survey, etc., that did not meet the criteria for the definition of pain persistence (incident pain). We further categorized the distribution of pain as follows: (1) no pain or single-site pain at baseline survey and follow-up survey; (2) a shift from no pain or single-site pain at baseline survey to multisite pain at follow-up survey; (3) multisite pain at both baseline survey and follow-up survey (multisite pain persistence); and (4) and a shift from multisite pain at baseline survey to no pain or single-site pain at follow-up survey.

### Other Measurements

At baseline, sociodemographic data were collected, encompassing age, gender, marital status (partnered, single), education level (illiterate, elementary, middle school, high school, and above), and residence area (rural, urban). Health status and functioning-related factors were also documented, including medical diagnoses such as hyperglycemia, hypertension, stroke, heart disease, chronic lung disease, asthma, liver disease, emotional and mental disorders, and malignancies, through face-to-face interviews. Participants’ drinking and smoking habits were categorized as ever/present or never. Additionally, to assess fall incidents, participants were asked, “Have you fallen in the last 2 years (yes or no)?”

### Statistical Analysis

Student’s *t* test, analysis of variance, chi-square tests, and Kruskal–Wallis test were used to examine the differences in sociodemographic, health status, and functional characteristics of the study population with different pain statuses. In addition, chi-square tests were also used to examine whether there were trends in the incidence of sarcopenia observed according to different pain characteristics over the 4-year follow-up.

We used logistic regression models to investigate the link between pain status (no pain, baseline pain) and pain distribution (no pain, single-site pain, multisite pain) at baseline and sarcopenia status at the 4-year follow-up. To account for potential confounding factors, 4 models were constructed, yielding odds ratios (ORs) and 95% confidence intervals (95% CIs) with varying covariate adjustments: Model 1 was unadjusted; Model 2 adjusted for gender and age; Model 3 further included residential area, education, and marital status; and Model 4 also incorporated smoking, drinking, comorbidities (hyperglycemia, hypertension, stroke, heart disease, chronic lung disease, asthma, liver disease, emotional and mental disorders, and malignancies), and falls. To investigate the association between pain characteristics over time and sarcopenia status, we used analytic methods similar to those used in other pain studies (25,26). We classified pain status into 3 categories: no pain, incident pain, and pain persistence, and used univariate and multivariate logistic regression models to analyze their association with sarcopenia. This allowed us to determine the association between pain status categories over time and sarcopenia. Similarly, we categorized the pain distribution into 4 categories: no pain or single-site pain at baseline survey and follow-up survey; a shift from no pain or single-site pain at baseline survey to multisite pain at follow-up survey; multisite pain persistence; and a shift from multisite pain at baseline survey to no pain or single-site pain at follow-up survey. As well, we analyzed the relationship between these 4 pain distribution categories and sarcopenia using univariate and multivariate logistic regression models to determine the association between changes in pain distribution categories over time and sarcopenia. In addition, to determine the relationship between pain characteristics and each subcomponent of sarcopenia, we submitted each subcomponent of sarcopenia separately as a dependent variable to the fully adjusted model (Model 4) for analysis. Each analysis was conducted after excluding participants with components at baseline survey (low grip strength: *n* = 433; SPPB score of ≤9: *n* = 1 063; low muscle mass: *n* = 235) to determine the association between pain characteristics and each subcomponent of sarcopenia. It should be noted that in all the above analyses, we adopted the automatic deletion method for the research objects with missing values of coordination variables.

To verify the robustness of the findings, we performed several sensitivity analyses. First, we used the method of multiple interpolation to fill in the missing values of the covariates and repeated all the analyses after filling in. Second, given that previous studies had pointed out the association between malignancy and diabetes and sarcopenia (7,27), we excluded participants who reported having malignancy and diabetes and then repeated all of the analyses. Finally, given the possible differences in response to pain between males and females, we analyzed males and females separately.

All analyses were performed using SPSS 26.0. A 2-tailed *p* value < .05 was considered statistically significant.

## Results

Table 1 outlines the baseline characteristics of the study participants segmented by pain status, identifying 3 671 participants (65.9%) with no pain and 1 897 participants (34.1%) with baseline pain. Compared to participants who reported no pain, those with baseline pain were more likely to be female, have lower education levels, reside in rural areas, be single, and engage in drinking and smoking. Significant differences were observed in comorbidity distributions between the 2 groups (*p* < .05), with the baseline pain group exhibiting lower muscle mass, lower SPPB scores, and lower grip strength. After 4 years of follow-up, participants were further categorized based on changes in pain status over time into the following groups: no pain group (*n* = 2 960), incident pain group (*n* = 1 750), and pain persistence (*n* = 858). Detailed characteristics of the participants based on the change in pain status category over time are provided in Supplementary Table 1.

**Table 1.** Baseline Participant Characteristics According to Pain Status

Characteristics	Total, *n* = 5 568	No pain, *n* = 3 671	Baseline pain, *n* = 1 897	*p* Value
Age (years, *M* ± SD)	59.83 ± 6.60	59.85 ± 6.60	59.78 ± 6.60	.680
Gender (*n*, %)				<.001
Male	2 703 (48.5)	1 969 (53.6)	734 (38.7)	
Female	2 865 (51.5)	1 702 (46.4)	1 163 (61.3)	
Residential area (*n*, %)^*^				<.001
Rural	4 576 (82.2)	2 907 (79.2)	1 669 (88.0)	
Urban	991 (17.8)	763 (20.8)	228 (12.0)	
Marital status (*n*, %)				.074
Partnered	4 985 (89.5)	3 306 (90.1)	1 679 (88.5)	
Single	583 (10.5)	365 (9.9)	218 (11.5)	
Educational level (*n*, %)				<.001
Illiterate	2 718 (48.8)	1 622 (44.2)	1 096 (57.8)	
Primary school	1 297 (23.3)	867 (23.6)	430 (22.7)	
Middle school	1 015 (18.2)	757 (20.6)	258 (13.6)	
High school or above	538 (9.7)	425 (11.6)	113 (6.0)	
Ever/current smoking (*n*, %)				<.001
No	3 319 (59.6)	2 090 (56.9)	1 229 (64.8)	
Yes	2 249 (40.4)	1 581 (43.1)	668 (35.2)	
Ever/current drinking (*n*, %)				<.001
No	3 768 (67.7)	2 417 (65.8)	1 351 (71.2)	
Yes	1 800 (32.3)	1 254 (34.2)	546 (28.8)	
Comorbidities (*n*, %)				
Hyperglycemia^*^	336 (6.1)	190 (5.2)	146 (7.8)	<.001
Hypertension^*^	1 462 (26.4)	904 (24.7)	558 (29.6)	<.001
Malignancies^*^	47 (0.8)	24 (0.7)	23 (1.2)	<.030
Asthma^*^	191 (3.4)	83 (2.3)	108 (5.7)	<.001
Stroke^*^	95 (1.7)	49 (1.3)	46 (2.4)	.003
Chronic lung diseases^*^	527 (9.5)	245 (6.7)	282 (14.9)	<.001
Heart disease^*^	649 (11.7)	327 (9.0)	322 (17.1)	<.001
Liver disease^*^	217 (3.9)	94 (2.6)	123 (6.5)	<.001
Emotional and mental disorders^*^	64 (1.2)	25 (0.7)	39 (2.1)	<.001
SPPB score	11 (10, 12)	11 (10, 12)	11 (9, 12)	<.001
Grip strength (kg, *M* ± SD)	32.4 ± 10.3	33.7 ± 10.6	30.0 ± 9.1	<.001
ASM/Ht^2^ (kg/m^2^, *M* ± SD)	6.9 ± 1.0	7.0 ± 1.0	6.7 ± 1.0	<.001
Fall in last 2 years (*n*, %)^*^	920 (16.6)	454 (12.4)	466 (24.6)	<.001

*Notes*: ASM = appendicular skeletal muscle; SD = standard deviation; SPPB = The Short Physical Performance Battery.

^*^Missing data: 1 for residential area, 54 for hyperglycemia, 24 for hypertension, 26 for malignancies, 22 for asthma, 19 for stroke, 20 for chronic lung diseases, 31 for heart disease, 43 for liver disease, 27 for emotional and mental disorders, and 13 for falls.

**Figure 1.**
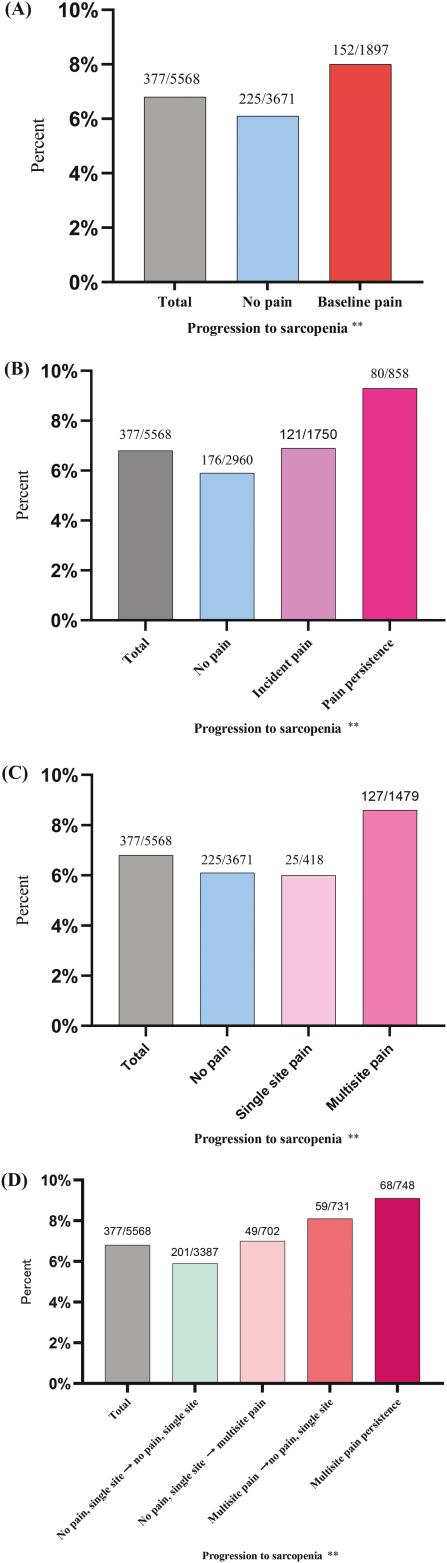
Occurrence of sarcopenia in 4 years according to (A) pain status at baseline, (B) change in pain status over time, (C) pain distribution at baseline, and (D) change in pain distribution over time. Chi-square test for trend ** (*p* < .01).

Figure 1 shows significant trends in the incidence of sarcopenia observed over the 4-year follow-up period based on different pain characteristics. After 4 years of follow-up, the overall incidence of sarcopenia was 6.8%. Based on pain status at baseline, the progression to sarcopenia after 4 years was 6.1% for no pain and 8.0% for baseline pain (*p* value for trend < .01, Figure 1A). According to pain distribution at baseline, the progression to sarcopenia after 4 years was 6.1% for no pain, 6.0% for single-site pain, and 8.6% for multisite pain (*p* value for trend < .01, Figure 1B). The progression to sarcopenia after 4 years was 5.9% for no pain, 6.9% for incident pain, and 9.3% for pain persistence (*p* value for trend < .01, Figure 1C), based on the change in pain status over time. The progression to sarcopenia after 4 years based on pain distribution over time was 5.9% for no pain or single-site pain at both the baseline survey and follow-up survey, 7.0% for a shift from no pain or single-site pain at the baseline survey to multisite pain at follow-up survey, 9.1% for multisite pain persistence, and 8.1% for a shift from multisite pain at baseline survey to no pain or single-site pain at follow-up survey (*p* value for trend < .01, Figure 1D).

Table 2 shows the association between pain status and pain distribution at baseline and sarcopenia at the 4-year follow-up using logistic regression models. In the unadjusted model (Model 1), participants with baseline pain or multisite pain were more likely to progress to sarcopenia than those without pain, with ORs of 1.33 (95% CI: 1.08–1.65) and 1.44 (95% CI: 1.15–1.80), respectively. Even after adjusting for other covariates (Models 2, 3, and 4), baseline pain and multisite pain remained significantly related to the progression of sarcopenia. However, single-site pain was not found to be significantly correlated to the progression of sarcopenia in either univariate or multivariate models.

**Table 2.** The Link Between Pain Status and Pain Distribution at Baseline and Sarcopenia Status at the 4-Year Follow-Up by Logistic Regression Models

Pain Characteristics	Model 1		Model 2		Model 3		Model 4	
	OR (95% CI)	*p* Value	OR (95% CI)	*p* Value	OR (95% CI)	*p* Value	OR (95% CI)	*p* Value
Pain status								
No pain	1 (Ref.)		1 (Ref.)		1 (Ref.)		1 (Ref.)	
Baseline pain	1.33 (1.08–1.65)	.008	1.39 (1.12–1.73)	.003	1.26 (1.01–1.57)	.040	1.28 (1.01–1.62)	.042
Pain distribution								
No pain	1 (Ref.)		1 (Ref.)		1 (Ref.)		1 (Ref.)	
Single-site pain	0.97 (0.64–1.49)	.905	1.01 (0.66–1.56)	.951	1.00 (0.65–1.54)	.984	0.99 (0.63–1.54)	.956
Multisite pain	1.44 (1.15–1.80)	.002	1.50 (1.19–1.89)	<.001	1.33 (1.05–1.69)	.017	1.37 (1.06–1.77)	.015

*Notes*: Model 1 was unadjusted.

Model 2 was adjusted for gender and age.

Model 3 was adjusted for gender, age, residential area, education, and marital status.

Model 4 was adjusted for gender, age, residential area, education, marital status, smoking, drinking, comorbidities (hyperglycemia, hypertension, stroke, heart disease, chronic lung disease, asthma, liver disease, emotional and mental disorders, and malignancies), and falls.

The odds ratios (ORs) and 95% confidence intervals (CIs) were obtained from univariate and multivariate logistic regression analyses.

Table 3 demonstrates the association between changes in pain status and pain distribution over time and the progression of sarcopenia using logistic regression models. Without covariate adjustment (Model 1), participants who reported pain persistence or multisite pain persistence had a greater likelihood of progressing to sarcopenia, with ORs of 1.63 (95% CI: 1.23–2.14) and 1.59 (95% CI: 1.19–2.11), respectively, compared to those without pain. These findings remained robust after adjusting for covariates in subsequent models (Models 2, 3, and 4). In both univariate and multivariate models, incident pain, a shift from no sites or single-site pain at the baseline survey to multisite pain at the follow-up survey, and a shift from multisite pain at the baseline survey to no pain or single-site pain at the follow-up survey were not correlated to the progression of sarcopenia.

**Table 3.** Associations Between Changes in Pain Status and Pain Distribution Over Time and Progression of Sarcopenia Status by Logistic Regression Models

Pain Characteristics	Model 1		Model 2		Model 3		Model 4	
	OR (95% CI)	*p* Value	OR (95% CI)	*p* Value	OR (95% CI)	*p* Value	OR (95% CI)	*p* Value
Change in pain status								
No pain	1 (Ref.)		1 (Ref.)		1 (Ref.)		1 (Ref.)	
Incident pain	1.17 (0.92–1.49)	.187	1.17 (0.92–1.50)	.197	1.05 (0.82–1.34)	.698	1.06 (0.81–1.37)	.685
Pain persistence	1.63 (1.23–2.14)	<.001	1.74 (1.31–2.32)	<.001	1.50 (1.13–2.00)	.005	1.61 (1.19–2.19)	.002
Change in pain distribution								
No pain, single-site to no pain, single-site	1 (Ref.)		1 (Ref.)		1 (Ref.)		1 (Ref.)	
No pain, single-site to multisite pain	1.19 (0.86–1.64)	.293	1.20 (0.87–1.68)	.268	1.08 (0.77–1.50)	.659	1.16 (0.83–1.63)	.380
Multisite pain to no pain, single-site	1.39 (1.03–1.88)	.032	1.43 (1.05–1.94)	.024	1.26 (0.92–1.72)	.144	1.29 (0.92–1.80)	.141
Multisite pain persistence	1.59 (1.19–2.11)	.002	1.68 (1.25–2.26)	<.001	1.45 (1.07–1.95)	.015	1.56 (1.13–2.14)	.006

*Notes*: Model 1 was unadjusted.

Model 2 was adjusted for gender and age.

Model 3 was adjusted for gender, age, residential area, education, and marital status.

Model 4 was adjusted for gender, age, residential area, education, marital status, smoking, drinking, comorbidities (hyperglycemia, hypertension, stroke, heart disease, chronic lung disease, asthma, liver disease, emotional and mental disorders, and malignancies), and falls.

The odds ratios (ORs) and 95% confidence intervals (CIs) were obtained from univariate and multivariate logistic regression analyses.

**Figure 2.**
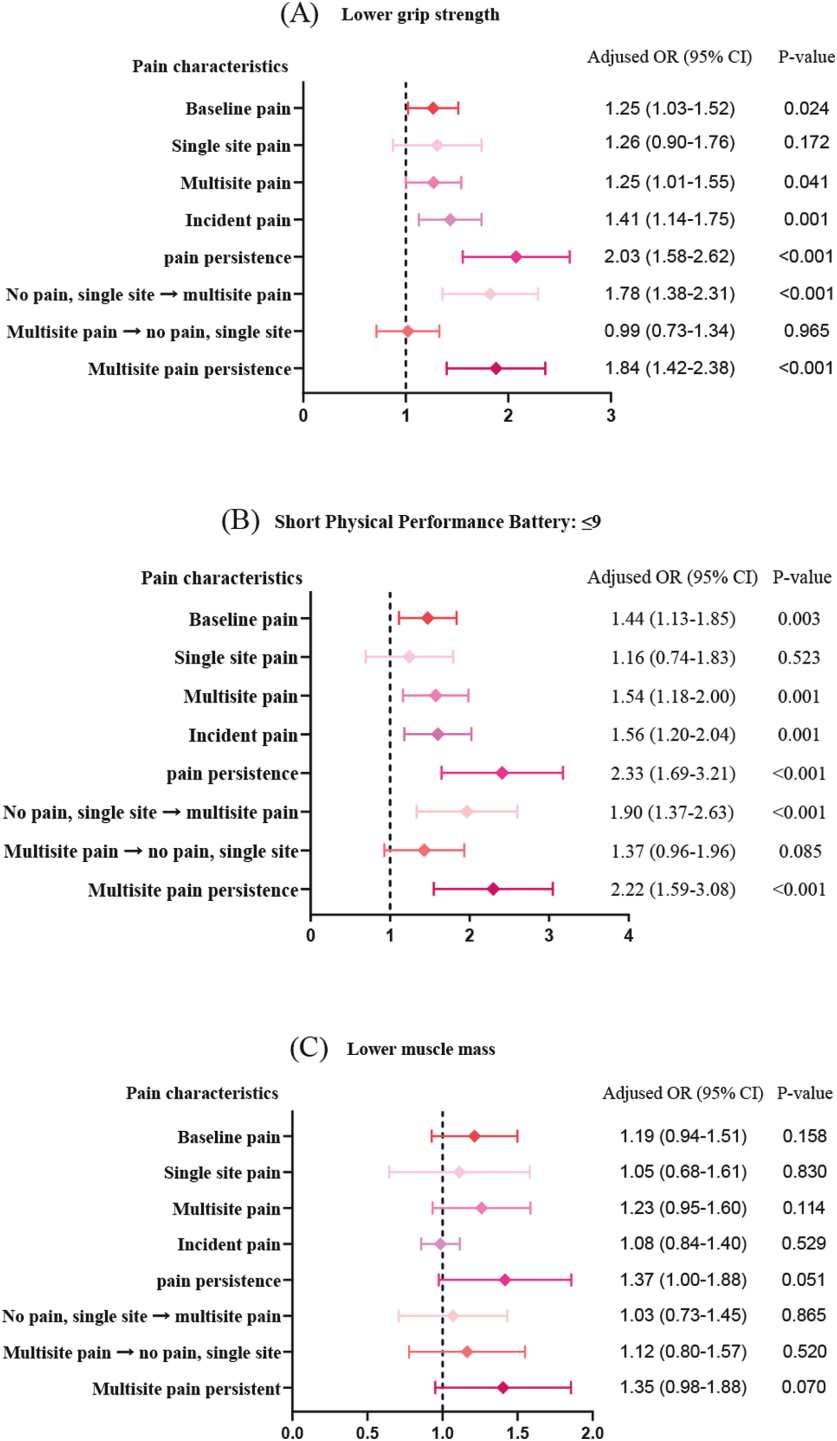
The link between pain characteristics and progression of sarcopenia subcomponents was analyzed by logistic regression models. Adjusted for age, sex, marital status, education level, residence area, hyperglycemia, hypertension, stroke, heart disease, chronic lung disease, asthma, liver disease, emotional and mental disorders, arthritis, malignancies, and falls (Model 4). Each analysis was conducted after excluding participants with the component of sarcopenia at baseline. CI = confidence interval; OR = odds ratio.

Regarding the progression of each subcomponent of sarcopenia, after adjusting for other covariates, our analysis revealed that baseline pain, multisite pain, pain persistence, and multisite pain persistence were significantly correlated to low grip strength, with ORs of 1.25 (95% CI: 1.03–1.52), 1.25 (95% CI: 1.01–1.55), 2.03 (95% CI: 1.58–2.62), and 1.84 (95% CI: 1.42–2.38), respectively (Figure 2A). In addition, baseline pain, multisite pain, pain persistence, and multisite pain persistence were also significantly correlated to clinically meaningful SPPB decline, with ORs of 1.44 (95% CI: 1.13–1.85), 1.54 (95% CI: 1.18–2.00), 2.33 (95% CI: 1.69–3.21), and 2.22 (95% CI: 1.59–3.08), respectively (Figure 2B). However, our analysis did not reveal a close correlation between pain and low muscle mass (Figure 2C).

Sensitivity analyses showed that the results did not change significantly after filling in the missing values using the multiple interpolation approach (Supplementary Tables 2, 3, and 4). Similar results were found after further exclusion of participants with malignant tumors and diabetes (Supplementary Tables 5, 6, and 7). In addition, the association between pain characteristics and the sarcopenia subcomponent was found to be consistent with the overall population when analyzed separately by males and females (Supplementary Tables 8 and 9). However, the association between pain characteristics and sarcopenia was statistically significant in male participants but not in female participants (Supplementary Tables 10, 11, 12, and 13).

## Discussion

In our study of a representative cohort of middle-aged and older Chinese adults, we observed that pain was correlated with the risk of progression to sarcopenia, especially when pain persisted at multiple body sites. This correlation remained significant even after accounting for multiple factors such as gender, age, education level, hyperglycemia, hypertension, stroke, heart disease, chronic lung disease, asthma, liver disease, emotional and mental disorders, malignancies, smoking, alcohol consumption, and falls. Moreover, our multivariate analyses revealed a significant link between pain and both low grip strength and clinically meaningful SPPB decline, independent of low muscle mass.

Sarcopenia is a kind of age-related disease (7). Due to accumulating evidence, it has been established that muscle mass tends to decrease by approximately 1% to 2% per year after the age of 50, accompanied by a decline in muscle strength ranging from 1.5% to 5.0% per year (1). Accordingly, we purposely selected people aged 50 years and older as the subjects of our study. Longitudinal analysis indicated that, compared to those free from pain, individuals experiencing pain had an elevated risk of sarcopenia progression. This result was consistent with 2 recently conducted longitudinal studies (15,16). In addition, based on the available evidence, we further added to the link between changes in pain characteristics over time and the progression to sarcopenia, as well as correlations between pain characteristics and the various subcomponents of sarcopenia. In terms of pain status, our study found that the risk of progression to sarcopenia in middle-aged and older adults was increased only when pain was persistent, whereas there was no significant association between incident pain and progression to sarcopenia. In terms of pain distribution, we only observed a strong correlation between multisite pain persistence and progression to sarcopenia in middle-aged and older adults. However, a shift from no pain or single-site pain at the baseline survey to multisite pain at the follow-up survey, as well as a shift from multisite pain at the baseline survey to no pain or single-site pain at the follow-up survey, showed no statistically significant association with the progression to sarcopenia. These findings suggested that pain may increase the risk of developing sarcopenia only when it reaches a certain cumulative amount in the body, and that the damage caused by pain may have temporal and spatial cumulative effects. Previous studies have also mentioned the temporal and spatial summation of pain (28,29). When localized areas are repetitively subjected to painful stimuli or when multiple sites experience pain simultaneously, pain will show a superimposed effect. For example, studies comparing the impact of acute pain and chronic pain on the body found that, unlike acute pain, chronic pain manifested pathologically and inadequately, causing harm to affected individuals (30). A study conducted in Germany found that the number of pain sites in community-dwelling older adults was associated with depression (31). Similarly, a UK study found that the number of pain sites was associated with low health-related quality of life in people over 50 years of age (32). Furthermore, in our analysis of various subcomponents of sarcopenia, we observed that pain was not correlated with every subcomponent of sarcopenia. Pain contributed to poorer physical performance and lower grip strength, but did not appear to be significantly correlated to loss of muscle mass. These findings contribute to a more comprehensive understanding of the effects of pain on the body and provide guidance for developing more targeted interventions.

It was important to note that when analyzed separately for males and females, we found that pain was associated with an increased risk of sarcopenia in middle-aged and older males, while there was no statistical significance between pain characteristics and sarcopenia in females. This gender-specific outcome aligns with Lin et al.’s findings to some extent (16). For the gender difference, we believed that this could be due to the different physiological and behavioral responses to pain in males and females (33). Recent studies have also described gender as a key factor affecting pain experience and coping strategies (34). Clinical studies have shown that there are significant differences in responses to pain between males and females (35,36). Females have lower thresholds and tolerance for evoked pain and they are more sensitive to pain compared to males (33). Some studies have suggested that this may be related to sex hormones, with estrogen and/or progesterone increasing susceptibility to pain and testosterone having a protective effect (37). In addition, when confronted with pain, males and females adopt different coping strategies (38). For example, males are more likely to take behavioral distractions and avoid reporting pain, whereas females are more likely to seek medical care and proactively report pain (38,39). Studies also have shown that compared to males, females tend to use more prescription medications to self-manage pain (40). Thus, some studies have shown that females are more likely to experience pain while showing that pain has a more profound effect on males (41). This gender difference also indirectly reflected the importance of identifying and developing timely pain interventions in clinical practice. It also suggested that clinicians should consider the influence of gender factors when developing pain relief programs.

Till now, it seems inconclusive whether there is an independent correlation between pain and sarcopenia. Although our study was unable to reveal a direct causal relationship between pain and sarcopenia, it confirmed a strong correlation between the two. Many possible explanations for the correlation between pain and sarcopenia can be considered. Firstly, they share common risk factors. The occurrence of pain and sarcopenia not only portends similar adverse outcomes but also shares many common risk factors such as increasing age, sedentary lifestyle, certain chronic diseases (eg, osteoarthritis, osteoporosis), and so on (7). Secondly, pain can serve as a clinical manifestation of sarcopenia. When sarcopenia occurs, the strength and integrity of skeletal muscles are compromised, potentially leading to joint instability, reduced joint mobility, and subsequently causing joint degeneration and pain (42). For example, the study by Han et al. found that sarcopenia was accompanied by increased occurrence of shoulder pain in community-dwelling older adults (43). The study by Sun et al. assessed the impact of sarcopenia on the quality of life in a study involving 4 937 older community patients in Korea. They found that individuals with sarcopenia have a higher possibility of experiencing pain (44). Thirdly, pain can act as a risk factor for sarcopenia. The presence of pain may lead middle-aged and older adults to restricted activity, sedentary behavior, and a fear of movement (8). Prolonged low-level physical activity leads to decreased muscle strength in the extremities and poorer physical performance, which in turn accelerates the onset and progression of sarcopenia (3). Additionally, the sustained presence of pain can keep the body in a state of chronic stress, leading to local or systemic inflammatory responses (45). Elevated levels of circulating inflammatory factors may directly impact the contractile force of skeletal muscles negatively (46). For example, research has shown that TNF-α and TNF-β can alter the formation and repair of the myelin sheath and motor neurons in muscle tissue, inducing functional defects and apoptosis of motor neurons, leading to a decline in limb muscle strength (46). The decline in muscle strength subsequently leads to deteriorating physical performance, disuse atrophy of skeletal muscles, and ultimately resulting in the onset and progression of sarcopenia. Lastly, certain factors may act as mediators between the two. Factors such as medications, comorbidities, and lifestyle factors can significantly influence the relationship between pain and sarcopenia (15,47). For example, opioids are strongly correlated with muscle loss. Excessive use of opioid analgesics by some pain patients may disrupt the balance between protein degradation and synthesis, leading to and exacerbating the progression of sarcopenia (48). Clinical studies have shown that chronic pain is often accompanied by comorbidities such as anxiety, depression, and sleep disorders (49), and those comorbidities are associated with an increased risk of developing sarcopenia (50,51). Overall, by gaining a deeper understanding of the relationship and the underlying mechanisms between the two, it will be beneficial for physicians and patients to recognize the importance of comprehensive screening for pain and sarcopenia. We can conclude that there are complex and close relationships between pain and sarcopenia. These findings suggested that in clinical practice we should not only prevent and intervene in the onset and progression of sarcopenia through pharmacologic management of pain but also pay attention to the use of nonpharmacologic treatments, especially muscle strength and physical exercise.

The strength of our study lies in the use of nationally representative data for longitudinal analysis. Based on existing evidence, our study fills the research gap on the link between pain characteristics over time and sarcopenia and also reveals the associations between pain characteristics and subcomponents of sarcopenia that have not been addressed in previous studies. However, there are several limitations in our study. Firstly, in the CHARLS study, the walking speed test was conditioned to require respondents to be 60 years of age and older. Given that walking speed is closely related to age, multiple studies on the relationship between age and walking speed have shown that the actual walking speed of individuals aged 50–59 often surpasses the 1.0 m/s threshold identified for slow walking speed (7,52,53). Therefore, in this study, we defaulted to normal walking speeds for participants under 60 years of age. Although we used the SPPB score to synthesize participants’ physical functional performance and to reduce the influence of walking speed testing on the overall assessment, this estimate may still bias the assessment of overall physical functional performance. Second, muscle mass in our study was estimated through a body measure formula previously validated in Chinese populations. This formula has not been validated in other racial populations (20). Considering the physiological and genetic differences between races, our results may be more applicable to Asian populations. Thirdly, the lack of medication-related information in our study hindered a comprehensive assessment of participants’ medication profiles. This limitation may have obscured the potential effects of medications on sarcopenia. For example, studies have shown that opioid analgesics, statin lipid-lowering drugs, and corticosteroids appear to have adverse effects on muscle mass (48,54). Thus, the lack of drug-related data may have significantly affected the results of our study. Future studies should consider the influence of medication on sarcopenia. Finally, our study only collected information on pain status and pain distribution but did not cover other pain characteristics, such as pain interference and pain severity, and their relationship with sarcopenia. Especially, the study on pain interference is still lacking. Therefore, further studies are needed to explore the effect of pain interference on sarcopenia status in middle-aged and older adults.

In conclusion, our study demonstrated that pain, especially pain persistence, was linked to an increased risk of progression to sarcopenia in Chinese middle-aged and older adults. Pain presence may decrease muscle strength, leading to poor physical performance, which in turn triggers the onset and progression of sarcopenia. These findings are beneficial for physicians and patients to recognize the importance of comprehensive screening for pain and sarcopenia. Furthermore, these findings also indicated that encouraging strength training in middle-aged and older adults to minimize the occurrence of pain-related complications may be beneficial in preventing and intervening in sarcopenia.

## Supplementary Material

glae080_suppl_Supplementary_Material
