# Supplementary material for: Pain Characteristics and Progression to Sarcopenia in Chinese Middle-Aged and Older Adults: A 4-Year Longitudinal Study
Source: J Gerontol A Biol Sci Med Sci. 2024 Mar 12;79(5):glae080. doi: 10.1093/gerona/glae080 (PMC11005774; doi:10.1093/gerona/glae080)
Supplement: glae080_suppl_Supplementary_Material [file glae080_suppl_supplementary_material.docx]

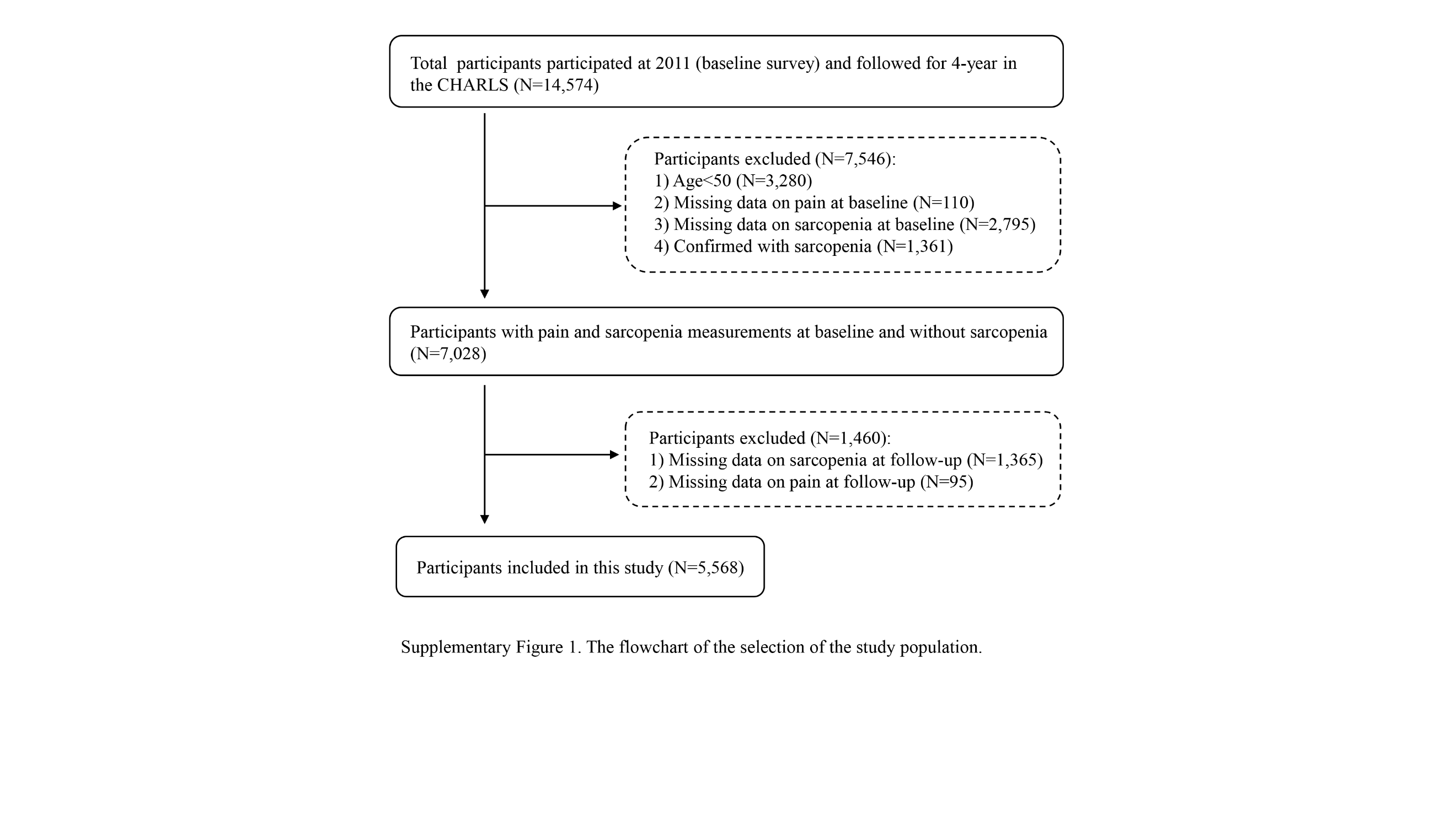


| Supplementary Table 1. Participant characteristics based on changes in pain status over time. | | | | |
| --- | --- | --- | --- | --- |
| Characteristics | No pain, n=2960 | Incident pain,  n=1750 | Pain persistence, n= 858 | P-value |
| Age (years, M ± SD) | 59.8 ± 6.5 | 59.9 ± 6.9 | 59.8 ±6.3 | 0.656 |
| Gender (n, %) |  |  |  | <0.001 |
| Male | 1663 (56.2) | 758 (43.3) | 282 (32.9) |  |
| Female | 1297 (43.8) | 992 (56.7) | 576 (67.1) |  |
| Residential area (n, %)^a^ |  |  |  | <0.001 |
| Rural | 2284 (77.2) | 1519 (86.8) | 773 (90.1) |  |
| Urban | 675 (22.8) | 231 (13.2) | 85 (9.9) |  |
| Marital status (n, %) |  |  |  | 0.074 |
| Partnered | 2678 (90.5) | 1556 (88.9) | 751 (87.5) |  |
| Single | 282 (9.5) | 194 (11.1) | 107 (12.5) |  |
| Educational level (n, %) |  |  |  | <0.001 |
| Illiterate | 1231 (41.6) | 945 (54.0) | 542 (63.2) |  |
| Primary school | 698 (23.6) | 414 (23.7) | 185 (21.6) |  |
| Middle school | 647 (21.9) | 276 (15.8) | 92 (10.7) |  |
| High school or above | 384 (13.0) | 115 (6.6) | 39 (4.5) |  |
| Ever/current smoking (n, %) |  |  |  | <0.001 |
| No | 1633 (55.2) | 1101 (62.9) | 585 (68.2) |  |
| Yes | 1327 (44.8) | 649 (37.1) | 273 (31.8) |  |
| Ever/current drinking (n, %) |  |  |  | <0.001 |
| No | 1897 (64.1) | 1239 (70.8) | 632 (73.7) |  |
| Yes | 1063 (35.9) | 511 (29.2) | 226 (26.3) |  |
| Co-morbidities (n, %) |  |  |  |  |
| Hyperglycemia^a^ | 155 (5.3) | 113 (6.5) | 68 (8.0) | 0.009 |
| Hypertension^a^ | 714 (24.2) | 482 (27.6) | 266 (31.1) | <0.001 |
| Malignancies^a^ | 23 (0.8) | 15 (0.9) | 9 (1.1) | 0.746 |
| Asthma^a^ | 70 (2.4) | 68 (3.9) | 53 (6.2) | <0.001 |
| Stroke^a^ | 35 (1.2) | 31 (1.8) | 29 (3.4) | <0.001 |
| Chronic lung diseases^a^ | 188 (6.4) | 200 (11.5) | 139 (16.3) | <0.001 |
| Heart disease^a^ | 239 (8.1) | 249 (14.3) | 161 (18.9) | <0.001 |
| Liver disease^a^ | 73 (2.5) | 83 (4.8) | 61 (7.2) | <0.001 |
| Emotional and mental disorders^a^ | 19 (0.6) | 30 (1.7) | 15 (1.8) | <0.001 |
| SPPB score | 11 (10, 12) | 11 (10, 12) | 11 (9, 12) | <0.001 |
| Grip strength (kg, M ± SD) | 34.3 ± 10.8 | 31.0 ± 9.3 | 28.6 ± 8.8 | <0.001 |
| ASM/Ht^2^ (kg/m^2^, M ± SD) | 7.0 ± 1.0 | 6.8 ± 1.0 | 6.6 ± 1.0 | <0.001 |
| Fall in last 2 years (n, %)^a^ | 353 (11.9) | 353 (20.2) | 214 (25.0) | <0.001 |

^a^Missing data: 1 for residential area, 54 for hyperglycemia, 24 for hypertension 26 for malignancies, 22 for asthma, 19 for stroke, 20 for chronic lung diseases, 31 for heart disease, 43 for liver disease, 27 for emotional and mental disorders, and 13 for falls.

SD: standard deviation, SPPB: The Short Physical Performance Battery, ASM: appendicular skeletal muscle.

| Supplementary Table 2. The link between pain status and pain distribution at baseline and sarcopenia at the 4-year follow-up by logistic regression models after filling in missing values of covariates using multiple interpolation. | | | | | | | | |
| --- | --- | --- | --- | --- | --- | --- | --- | --- |
| Pain characteristics | Model 1 | | Model 2 | | Model 3 | | Model 4 | |
|  | OR (95% CI) | P | OR (95% CI) | P | OR (95% CI) | P | OR (95% CI) | P |
| Pain status |  |  |  |  |  |  |  |  |
| No pain | 1 (Ref.) |  | 1 (Ref.) |  | 1 (Ref.) |  | 1 (Ref.) |  |
| Baseline pain | 1.33 (1.08-1.65) | 0.008 | 1.39 (1.12-1.73) | 0.003 | 1.26 (1.01-1.57) | 0.040 | 1.29 (1.03-1.63) | 0.028 |
| Pain distribution |  |  |  |  |  |  |  |  |
| No pain | 1 (Ref.) |  | 1 (Ref.) |  | 1 (Ref.) |  | 1 (Ref.) |  |
| Single site pain | 0.97 (0.64-1.49) | 0.905 | 1.01 (0.66-1.56) | 0.951 | 1.00 (0.65-1.54) | 0.985 | 0.99 (0.64-1.54) | 0.974 |
| Multisite pain | 1.44 (1.15-1.80) | 0.002 | 1.50 (1.19-1.89) | <0.001 | 1.33 (1.05-1.69) | 0.016 | 1.39 (1.09-1.78) | 0.009 |

Model 1 was unadjusted.

Model 2 was adjusted for gender and age.

Model 3 was adjusted for gender, age, residential area, education, and marital status.

Model 4 was adjusted for gender, age, residential area, education, marital status, smoking, drinking, co-morbidities (hyperglycemia, hypertension, stroke, heart disease, chronic lung disease, asthma, liver disease, emotional and mental disorders, and malignancies), and falls.

The odds ratios (ORs) and 95% confidence intervals (CIs) were obtained from univariate and multivariate logistic regression analyses.

| Supplementary Table 3. The link between changes in pain status and pain distribution over time and progression to sarcopenia by logistic regression models after filling in missing values of covariates using multiple interpolation. | | | | | | | | |
| --- | --- | --- | --- | --- | --- | --- | --- | --- |
| Pain characteristics | Model 1 | | Model 2 | | Model 3 | | Model 4 | |
|  | OR (95% CI) | P | OR (95% CI) | P | OR (95% CI) | P | OR (95% CI) | P |
| Change in pain status |  |  |  |  |  |  |  |  |
| No pain | 1 (Ref.) |  | 1 (Ref.) |  | 1 (Ref.) |  | 1 (Ref.) |  |
| Incident pain | 1.17 (0.92-1.49) | 0.187 | 1.17 (0.92-1.50) | 0.197 | 1.05 (0.82-1.34) | 0.696 | 1.06 (0.83-1.37) | 0.640 |
| pain persistence | 1.63 (1.23-2.14) | <0.001 | 1.74 (1.31-2.32) | <0.001 | 1.50 (1.13-2.00) | 0.005 | 1.57 (1.16-2.12) | 0.003 |
| Change in pain distribution |  |  |  |  |  |  |  |  |
| No pain, single site to no pain, single site | 1 (Ref.) |  | 1 (Ref.) |  | 1 (Ref.) |  | 1 (Ref.) |  |
| No pain, single site to multisite pain | 1.19 (0.86-1.64) | 0.293 | 1.20 (0.87-1.68) | 0.268 | 1.08 (0.77-1.50) | 0.657 | 1.09 (0.78-1.53) | 0.615 |
| Multisite pain to no pain, single site | 1.39 (1.03-1.88) | 0.032 | 1.43 (1.05-1.94) | 0.024 | 1.26 (0.92-1.72) | 0.144 | 1.32 (0.96-1.82) | 0.090 |
| Multisite pain persistence | 1.59 (1.19-2.11) | 0.002 | 1.68 (1.25-2.26) | <0.001 | 1.45 (1.07-1.95) | 0.015 | 1.51 (1.11-2.07) | 0.009 |

Model 1 was unadjusted.

Model 2 was adjusted for gender and age.

Model 3 was adjusted for gender, age, residential area, education, and marital status.

Model 4 was adjusted for gender, age, residential area, education, marital status, smoking, drinking, co-morbidities (hyperglycemia, hypertension, stroke, heart disease, chronic lung disease, liver disease, emotional and mental disorders, malignancies, and asthma), and falls.

The odds ratios (ORs) and 95% confidence intervals (CIs) were obtained from univariate and multivariate logistic regression analyses.

| Supplementary Table 4. The link between pain characteristics and each subcomponent of sarcopenia by logistic regression models after filling in missing values of covariates using multiple interpolation. | | | | | | |
| --- | --- | --- | --- | --- | --- | --- |
| Pain characteristics | Lower grip strength | | SPPB≤9 | | Lower muscle mass | |
|  | Adjust OR (95% CI) | P | Adjust OR (95% CI) | P | Adjust OR (95% CI) | P |
| Pain status |  |  |  |  |  |  |
| No pain | 1 (Ref.) |  | 1 (Ref.) |  | 1 (Ref.) |  |
| Baseline pain | 1.28 (1.05-1.55) | 0.013 | 1.47 (1.15-1.87) | 0.002 | 1.19 (0.94-1.50) | 0.151 |
| No pain | 1 (Ref.) |  | 1 (Ref.) |  | 1 (Ref.) |  |
| Incident pain | 1.40 (1.13-1.72) | 0.002 | 1.54 (1.19-2.00) | <0.001 | 1.06 (0.82-1.35) | 0.671 |
| pain persistence | 2.06 (1.61-2.64) | <0.001 | 2.34 (1.71-3.21) | 0.001 | 1.33 (0.97-1.82) | 0.072 |
| Pain distribution |  |  |  |  |  |  |
| No pain | 1 (Ref.) |  | 1 (Ref.) |  | 1 (Ref.) |  |
| Single site pain | 1.22 (0.88-1.70) | 0.234 | 1.24 (0.80-1.92) | 0.337 | 1.01 (0.66-1.56) | 0.949 |
| Multisite pain | 1.29 (1.05-1.60) | 0.016 | 1.54 (1.18-2.00) | 0.001 | 1.24 (0.96-1.60) | 0.094 |
| No pain, single site to no pain, single site | 1 (Ref.) |  | 1 (Ref.) |  | 1 (Ref.) |  |
| No pain, single site to multisite pain | 1.74 (1.35-2.25) | <0.001 | 1.87 (1.35-2.58) | <0.001 | 0.98 (0.69-1.37) | 0.886 |
| Multisite pain to no pain, single site | 1.06 (0.79-1.41) | 0.712 | 1.35 (0.95-1.92) | 0.091 | 1.14 (0.82-1.59) | 0.427 |
| Multisite pain persistence | 1.86 (1.44-2.41) | <0.001 | 2.20 (1.59-3.04) | <0.001 | 1.33 (0.96-1.83) | 0.083 |

Adjusted for gender, age, residential area, education, marital status, smoking, drinking, co-morbidities (hyperglycemia, hypertension, stroke, heart disease, chronic lung disease, liver disease, emotional and mental disorders, malignancies, and asthma), and falls (Model 4).

The odds ratios (ORs) and 95% confidence intervals (CIs) were obtained from multivariate logistic regression analyses.

| Supplementary Table 5. The link between pain status and pain distribution at baseline and sarcopenia status at the 4-year follow-up by logistic regression models after exclusion of participants with malignancy and diabetes. | | | | | | | | |
| --- | --- | --- | --- | --- | --- | --- | --- | --- |
| Pain characteristics | Model 1 | | Model 2 | | Model 3 | | Model 4 | |
|  | OR (95% CI) | P | OR (95% CI) | P | OR (95% CI) | P | OR (95% CI) | P |
| Pain status |  |  |  |  |  |  |  |  |
| No pain | 1 (Ref.) |  | 1 (Ref.) |  | 1 (Ref.) |  | 1 (Ref.) |  |
| Baseline pain | 1.31 (1.05-1.63) | 0.017 | 1.36 (1.08-1.70) | 0.008 | 1.25 (0.99-1.56) | 0.057 | 1.26 (1.00-1.60) | 0.053 |
| Pain distribution |  |  |  |  |  |  |  |  |
| No pain | 1 (Ref.) |  | 1 (Ref.) |  | 1 (Ref.) |  | 1 (Ref.) |  |
| Single site pain | 0.96 (0.62-1.48) | 0.843 | 1.00 (0.64-1.55) | 0.993 | 0.99 (0.64-1.54) | 0.966 | 0.99 (0.64-1.54) | 0.974 |
| Multisite pain | 1.41 (1.12-1.78) | 0.004 | 1.47 (1.16-1.87) | 0.002 | 1.32 (1.04-1.68) | 0.025 | 1.39 (1.09-1.78) | 0.009 |

Model 1 was unadjusted.

Model 2 was adjusted for gender and age.

Model 3 was adjusted for gender, age, residential area, education, and marital status.

Model 4 was adjusted for gender, age, residential area, education, marital status, smoking, drinking, co-morbidities (hypertension, stroke, heart disease, chronic lung disease, liver disease, emotional and mental disorders, and asthma), and falls.

The odds ratios (ORs) and 95% confidence intervals (CIs) were obtained from univariate and multivariate logistic regression analyses.

| Supplementary Table 6. The link between changes in pain status and pain distribution over time and progression to sarcopenia by logistic regression models after exclusion of participants with malignancy and diabetes. | | | | | | | | |
| --- | --- | --- | --- | --- | --- | --- | --- | --- |
| Pain characteristics | Model 1 | | Model 2 | | Model 3 | | Model 4 | |
|  | OR (95% CI) | P | OR (95% CI) | P | OR (95% CI) | P | OR (95% CI) | P |
| Change in pain status |  |  |  |  |  |  |  |  |
| No pain | 1 (Ref.) |  | 1 (Ref.) |  | 1 (Ref.) |  | 1 (Ref.) |  |
| Incident pain | 1.16 (0.91-1.48) | 0.244 | 1.16 (0.90-1.49) | 0.245 | 1.05 (0.81-1.35) | 0.719 | 1.05 (0.81-1.36) | 0.689 |
| pain persistence | 1.59 (1.19-2.11) | 0.001 | 1.70 (1.26-2.28) | <0.001 | 1.48 (1.10-1.99) | 0.009 | 1.52 (1.12-2.07) | 0.007 |
| Change in pain distribution |  |  |  |  |  |  |  |  |
| No pain, single site to no pain, single site | 1 (Ref.) |  | 1 (Ref.) |  | 1 (Ref.) |  | 1 (Ref.) |  |
| No pain, single site to multisite pain | 1.20 (0.86-1.66) | 0.279 | 1.22 (0.87-1.70) | 0.251 | 1.10 (0.78-1.53) | 0.593 | 1.10 (0.79-1.55) | 0.569 |
| Multisite pain to no pain, single site | 1.35 (0.99-1.85) | 0.058 | 1.40 (1.01-1.92) | 0.040 | 1.24 (0.90-1.72) | 0.184 | 1.29 (0.92-1.80) | 0.135 |
| Multisite pain persistence | 1.58 (1.17-2.12) | 0.003 | 1.66 (1.22-2.25) | 0.001 | 1.45 (1.06-1.97) | 0.019 | 1.49 (1.08-2.05) | 0.015 |

Model 1 was unadjusted.

Model 2 was adjusted for gender and age.

Model 3 was adjusted for gender, age, residential area, education, and marital status.

Model 4 was adjusted for gender, age, residential area, education, marital status, smoking, drinking, co-morbidities (hypertension, stroke, heart disease, chronic lung disease, liver disease, emotional and mental disorders, and asthma), and falls.

The odds ratios (ORs) and 95% confidence intervals (CIs) were obtained from univariate and multivariate logistic regression analyses.

| Supplementary Table 7. The link between pain characteristics and each subcomponent of sarcopenia by logistic regression models after exclusion of participants with malignancy and diabetes. | | | | | | |
| --- | --- | --- | --- | --- | --- | --- |
| Pain characteristics | Lower grip strength | | SPPB≤9 | | Lower muscle mass | |
|  | Adjust OR (95% CI) | P | Adjust OR (95% CI) | P | Adjust OR (95% CI) | P |
| Pain status |  |  |  |  |  |  |
| No pain | 1 (Ref.) |  | 1 (Ref.) |  | 1 (Ref.) |  |
| Baseline pain | 1.28 (1.05-1.56) | 0.015 | 1.42 (1.10-1.83) | 0.007 | 1.15 (0.90-1.46) | 0.267 |
| No pain | 1 (Ref.) |  | 1 (Ref.) |  | 1 (Ref.) |  |
| Incident pain | 1.39 (1.12-1.72) | 0.003 | 1.44 (1.10-1.90) | 0.009 | 1.06 (0.82-1.37) | 0.643 |
| pain persistence | 2.09 (1.62-2.70) | <0.001 | 2.37 (1.71-3.28) | <0.001 | 1.24 (0.90-1.73) | 0.189 |
| Pain distribution |  |  |  |  |  |  |
| No pain | 1 (Ref.) |  | 1 (Ref.) |  | 1 (Ref.) |  |
| Single site pain | 1.17 (0.83-1.66) | 0.365 | 1.33 (0.85-2.06) | 0.208 | 1.01 (0.65-1.57) | 0.951 |
| Multisite pain | 1.32 (1.06-1.63) | 0.013 | 1.45 (1.10-1.91) | 0.008 | 1.19 (0.91-1.55) | 0.195 |
| No pain, single site to no pain, single site | 1 (Ref.) |  | 1 (Ref.) |  | 1 (Ref.) |  |
| No pain, single site to multisite pain | 1.67 (1.28-2.18) | <0.001 | 1.86 (1.34-2.59) | <0.001 | 0.99 (0.70-1.40) | 0.975 |
| Multisite pain to no pain, single site | 1.04 (0.77-1.41) | 0.792 | 1.13 (0.77-1.67) | 0.521 | 1.14 (0.81-1.61) | 0.441 |
| Multisite pain persistence | 1.92 (1.47-2.50) | <0.001 | 2.22 (1.58-3.10) | <0.001 | 1.23 (0.88-1.73) | 0.228 |

Adjusted for gender, age, residential area, education, marital status, smoking, drinking, co-morbidities (hypertension, stroke, heart disease, chronic lung disease, liver disease, emotional and mental disorders, and asthma), and falls.

The odds ratios (ORs) and 95% confidence intervals (CIs) were obtained from multivariate logistic regression.

| Supplementary Table 8. The link between pain characteristics and each subcomponent of sarcopenia by logistic regression models in male participants. | | | | | | |
| --- | --- | --- | --- | --- | --- | --- |
| Pain characteristics | Lower grip strength | | SPPB≤9 | | Lower muscle mass | |
|  | Adjust OR (95% CI) | P | Adjust OR (95% CI) | P | Adjust OR (95% CI) | P |
| Pain status |  |  |  |  |  |  |
| No pain | 1 (Ref.) |  | 1 (Ref.) |  | 1 (Ref.) |  |
| Baseline pain | 1.34 (1.00-1.79) | 0.054 | 1.29 (0.86-1.93) | 0.218 | 1.23 (0.90-1.69) | 0.195 |
| No pain | 1 (Ref.) |  | 1 (Ref.) |  | 1 (Ref.) |  |
| Incident pain | 1.41 (1.03-1.91) | 0.029 | 1.20 (0.79-1.83) | 0.394 | 1.01 (0.72-1.40) | 0.965 |
| pain persistence | 2.40 (1.63-3.54) | <0.001 | 2.29 (1.36-3.87) | 0.002 | 1.61 (1.04-2.48) | 0.033 |
| Pain distribution |  |  |  |  |  |  |
| No pain | 1 (Ref.) |  | 1 (Ref.) |  | 1 (Ref.) |  |
| Single site pain | 1.00 (0.58-1.72) | 0.998 | 0.61 (0.24-1.56) | 0.306 | 0.85 (0.46-1.58) | 0.609 |
| Multisite pain | 1.46 (1.06-2.02) | 0.020 | 1.54 (1.00-2.38) | 0.049 | 1.38 (0.98-1.95) | 0.069 |
| No pain, single site to no pain, single site | 1 (Ref.) |  | 1 (Ref.) |  | 1 (Ref.) |  |
| No pain, single site to multisite pain | 1.63 (1.08-2.45) | 0.019 | 1.61 (0.92-2.81) | 0.095 | 1.05 (0.66-1.68) | 0.832 |
| Multisite pain to no pain, single site | 1.14 (0.74-1.77) | 0.557 | 1.27 (0.71-2.27) | 0.419 | 1.18 (0.75-1.85) | 0.483 |
| Multisite pain persistence | 2.22 (1.48-3.33) | <0.001 | 2.41 (1.39-4.16) | 0.002 | 1.71 (1.10-2.68) | 0.018 |

Adjusted for age, residential area, education, marital status, smoking, drinking, co-morbidities (hyperglycemia, hypertension, stroke, heart disease, chronic lung disease, asthma, liver disease, emotional and mental disorders, and malignancies), and falls.

The odds ratios (ORs) and 95% confidence intervals (CIs) were obtained from multivariate logistic regression.

| Supplementary Table 9. The link between pain characteristics and each subcomponent of sarcopenia by logistic regression models in female participants. | | | | | | |
| --- | --- | --- | --- | --- | --- | --- |
| Pain characteristics | Lower grip strength | | SPPB≤9 | | Lower muscle mass | |
|  | Adjust OR (95% CI) | P | Adjust OR (95% CI) | P | Adjust OR (95% CI) | P |
| Pain status |  |  |  |  |  |  |
| No pain | 1 (Ref.) |  | 1 (Ref.) |  | 1 (Ref.) |  |
| Baseline pain | 1.21 (0.94-1.57) | 0.145 | 1.61 (1.18-2.19) | 0.003 | 1.12 (0.80-1.58) | 0.515 |
| No pain | 1 (Ref.) |  | 1 (Ref.) |  | 1 (Ref.) |  |
| Incident pain | 1.37 (1.03-1.83) | 0.032 | 1.86 (1.32-2.63) | <0.001 | 1.10 (0.75-1.59) | 0.628 |
| pain persistence | 1.86 (1.34-2.58) | <0.001 | 2.51 (1.68-3.76) | <0.001 | 1.12 (0.71-1.75) | 0.623 |
| Pain distribution |  |  |  |  |  |  |
| No pain | 1 (Ref.) |  | 1 (Ref.) |  | 1 (Ref.) |  |
| Single site pain | 1.37 (1.03-1.83) | 0.032 | 1.71 (1.02-2.86) | 0.043 | 1.19 (0.66-2.16) | 0.563 |
| Multisite pain | 1.86 (1.34-2.58) | <0.001 | 1.58 (1.13-2.20) | 0.007 | 1.10 (0.76-1.60) | 0.610 |
| No pain, single site to no pain, single site | 1 (Ref.) |  | 1 (Ref.) |  | 1 (Ref.) |  |
| No pain, single site to multisite pain | 1.79 (1.29-2.50) | 0.001 | 2.05 (1.37-3.07) | <0.001 | 0.87 (0.53-1.43) | 0.572 |
| Multisite pain to no pain, single site | 0.96 (0.65-1.43) | 0.849 | 1.42 (0.91-2.23) | 0.122 | 1.07 (0.66-1.73) | 0.781 |
| Multisite pain persistence | 1.65 (1.18-2.31) | 0.003 | 2.16 (1.44-3.24) | <0.001 | 1.02 (0.64-1.62) | 0.943 |

Adjusted for age, residential area, education, marital status, smoking, drinking, co-morbidities (hyperglycemia, hypertension, stroke, heart disease, chronic lung disease, asthma, liver disease, emotional and mental disorders, and malignancies), and falls.

The odds ratios (ORs) and 95% confidence intervals (CIs) were obtained from multivariate logistic regression.

| Supplementary Table 10. The link between pain status and pain distribution at baseline and sarcopenia status at the 4-year follow-up by logistic regression models in male participants. | | | | | | | | |
| --- | --- | --- | --- | --- | --- | --- | --- | --- |
| Pain characteristics | Model 1 | | Model 2 | | Model 3 | | Model 4^*^ | |
|  | OR (95% CI) | P | OR (95% CI) | P | OR (95% CI) | P | OR (95% CI) | P |
| Pain status |  |  |  |  |  |  |  |  |
| No pain | 1 (Ref.) |  | 1 (Ref.) |  | 1 (Ref.) |  | 1 (Ref.) |  |
| Baseline pain | 1.70 (1.26-2.30) | <0.001 | 1.71 (1.26-2.31) | 0.001 | 1.49 (1.09-2.02) | 0.011 | 1.49 (1.08-2.05) | 0.016 |
| Pain distribution |  |  |  |  |  |  |  |  |
| No pain | 1 (Ref.) |  | 1 (Ref.) |  | 1 (Ref.) |  | 1 (Ref.) |  |
| Single site pain | 0.77 (0.39-1.55) | 0.465 | 0.76 (0.38-1.53) | 0.437 | 0.74 (0.37-1.50) | 0.405 | 0.71 (0.35-1.45) | 0.350 |
| Multisite pain | 2.03 (1.49-2.78) | <0.001 | 2.06 (1.49-2.83) | <0.001 | 1.73 (1.25-2.40) | 0.001 | 1.79 (1.27-2.52) | 0.001 |

Model 1 was unadjusted.

Model 2 was adjusted for age.

Model 3 was adjusted for age, residential area, education, and marital status.

Model 4 was adjusted for age, residential area, education, marital status, smoking, drinking, co-morbidities (hyperglycemia, hypertension, stroke, heart disease, chronic lung disease, asthma, liver disease, emotional and mental disorders, and malignancies), and falls.

The odds ratios (ORs) and 95% confidence intervals (CIs) were obtained from univariate and multivariate logistic regression analyses.

| Supplementary Table 11. The link between changes in pain status and pain distribution over time and progression to sarcopenia by logistic regression models in male participants. | | | | | | | | |
| --- | --- | --- | --- | --- | --- | --- | --- | --- |
| Pain characteristics | Model 1 | | Model 2 | | Model 3 | | Model 4 | |
|  | OR (95% CI) | P | OR (95% CI) | P | OR (95% CI) | P | OR (95% CI) | P |
| Change in pain status |  |  |  |  |  |  |  |  |
| No pain | 1 (Ref.) |  | 1 (Ref.) |  | 1 (Ref.) |  | 1 (Ref.) |  |
| Incident pain | 1.33 (0.95-1.85) | 0.092 | 1.28 (0.91-1.79) | 0.153 | 1.09 (0.77-1.53) | 0.632 | 1.08 (0.76-1.54) | 0.661 |
| pain persistence | 2.56 (1.73-3.78) | <0.001 | 2.73 (1.83-4.06) | <0.001 | 2.20 (1.47-3.29) | <0.001 | 2.24 (1.47-3.42) | <0.001 |
| Change in pain distribution |  |  |  |  |  |  |  |  |
| No pain, single site to no pain, single site | 1 (Ref.) |  | 1 (Ref.) |  | 1 (Ref.) |  | 1 (Ref.) |  |
| No pain, single site to multisite pain | 1.32 (0.82-2.11) | 0.259 | 1.35 (0.83-2.18) | 0.222 | 1.14 (0.70-1.85) | 0.605 | 1.16 (0.71-1.90) | 0.559 |
| Multisite pain to no pain, single site | 1.75 (1.15-2.66) | 0.008 | 1.73 (1.13-2.65) | 0.011 | 1.47 (0.96-2.27) | 0.079 | 1.53 (0.97-2.40) | 0.065 |
| Multisite pain persistence | 2.70 (1.80-4.03) | <0.001 | 2.82 (1.88-4.25) | <0.001 | 2.26 (1.49-3.43) | <0.001 | 2.35 (1.52-3.65) | <0.001 |

Model 1 was unadjusted.

Model 2 was adjusted for age.

Model 3 was adjusted for age, residential area, education, and marital status.

Model 4 was adjusted for age, residential area, education, marital status, smoking, drinking, co-morbidities (hyperglycemia, hypertension, stroke, heart disease, chronic lung disease, asthma, liver disease, emotional and mental disorders, and malignancies), and falls.

The odds ratios (ORs) and 95% confidence intervals (CIs) were obtained from univariate and multivariate logistic regression analyses.

| Supplementary Table 12. The link between pain status and pain distribution at baseline and sarcopenia status at the 4-year follow-up by logistic regression models in female participants. | | | | | | | | |
| --- | --- | --- | --- | --- | --- | --- | --- | --- |
| Pain characteristics | Model 1 | | Model 2 | | Model 3 | | Model 4^*^ | |
|  | OR (95% CI) | P | OR (95% CI) | P | OR (95% CI) | P | OR (95% CI) | P |
| Pain status |  |  |  |  |  |  |  |  |
| No pain | 1 (Ref.) |  | 1 (Ref.) |  | 1 (Ref.) |  | 1 (Ref.) |  |
| Baseline pain | 1.12 (0.82-1.52) | 0.471 | 1.12 (0.82-1.53) | 0.471 | 1.06 (0.77-1.45) | 0.724 | 1.11 (0.80-1.54) | 0.547 |
| Pain distribution |  |  |  |  |  |  |  |  |
| No pain | 1 (Ref.) |  | 1 (Ref.) |  | 1 (Ref.) |  | 1 (Ref.) |  |
| Single site pain | 1.16 (0.67-2.00) | 0.595 | 1.21 (0.70-2.11) | 0.492 | 1.19 (0.68-2.08) | 0.541 | 1.22 (0.69-2.14) | 0.494 |
| Multisite pain | 1.11 (0.80-1.54) | 0.536 | 1.10 (0.79-1.54) | 0.580 | 1.03 (0.73-1.44) | 0.874 | 1.08 (0.75-1.54) | 0.683 |

Model 1 was unadjusted.

Model 2 was adjusted for age.

Model 3 was adjusted for age, residential area, education, and marital status.

Model 4 was adjusted for age, residential area, education, marital status, smoking, drinking, co-morbidities (hyperglycemia, hypertension, stroke, heart disease, chronic lung disease, asthma, liver disease, emotional and mental disorders, and malignancies), and falls.

The odds ratios (ORs) and 95% confidence intervals (CIs) were obtained from univariate and multivariate logistic regression analyses.

| Supplementary Table 13. The link between changes in pain status and pain distribution over time and progression to sarcopenia by logistic regression models in female participants. | | | | | | | | |
| --- | --- | --- | --- | --- | --- | --- | --- | --- |
| Pain characteristics | Model 1 | | Model 2 | | Model 3 | | Model 4 | |
|  | OR (95% CI) | P | OR (95% CI) | P | OR (95% CI) | P | OR (95% CI) | P |
| Pain status |  |  |  |  |  |  |  |  |
| No pain | 1 (Ref.) |  | 1 (Ref.) |  | 1 (Ref.) |  | 1 (Ref.) |  |
| Incident pain | 1.07 (0.75-1.51) | 0.713 | 1.03 (0.72-1.46) | 0.884 | 0.97 (0.68-1.39) | 0.889 | 1.01 (0.70-1.45) | 0.954 |
| pain persistence | 1.22 (0.82-1.81) | 0.334 | 1.17 (0.78-1.74) | 0.456 | 1.08 (0.72-1.61) | 0.723 | 1.15 (0.75-1.75) | 0.524 |
| Pain distribution |  |  |  |  |  |  |  |  |
| No pain, single site to no pain, single site | 1 (Ref.) |  | 1 (Ref.) |  | 1 (Ref.) |  | 1 (Ref.) |  |
| No pain, single site to multisite pain | 1.11 (0.71-1.73) | 0.643 | 1.02 (0.65-1.60) | 0.931 | 0.97 (0.62-1.53) | 0.909 | 0.99 (0.62-1.56) | 0.954 |
| Multisite pain to no pain, single site | 1.14 (0.74-1.77) | 0.557 | 1.12 (0.72-1.75) | 0.607 | 1.04 (0.67-1.63) | 0.847 | 1.09 (0.69-1.73) | 0.710 |
| Multisite pain persistence | 1.09 (0.72-1.66) | 0.676 | 1.04 (0.68-1.59) | 0.858 | 0.96 (0.63-1.47) | 0.855 | 1.01 (0.64-1.57) | 0.977 |

Model 1 was unadjusted.

Model 2 was adjusted for age.

Model 3 was adjusted for age, residential area, education, and marital status.

Model 4 was adjusted for age, residential area, education, marital status, smoking, drinking, co-morbidities (hyperglycemia, hypertension, stroke, heart disease, chronic lung disease, asthma, liver disease, emotional and mental disorders, and malignancies), and falls.

The odds ratios (ORs) and 95% confidence intervals (CIs) were obtained from univariate and multivariate logistic regression analyses.
